# The peptide derived from the Ig-like domain of human herpesvirus 8 K1 protein induces death in hematological cancer cells

**DOI:** 10.1186/1756-9966-31-69

**Published:** 2012-08-28

**Authors:** Urszula Daniluk, Celine Kerros, Rong-Hua Tao, Jillian F Wise, Xue Ao, Zuzana Berkova, Felipe Samaniego

**Affiliations:** 1 Department of Lymphoma and Myeloma, The University of Texas MD Anderson Cancer Center, 1515 Holcombe Blvd, Houston, TX, 77030, USA; 2 Department of Pediatrics, Gastroenterology and Allergology, Medical University of Bialystok, ul. Waszyngtona 17, Bialystok, 15-274, Poland; 3 Department of Immunology, The University of Texas MD Anderson Cancer Center, 1515 Holcombe Blvd, Houston, TX, 77030, USA

## Abstract

**Background:**

Although significant progress has been made in the treatment of lymphomas, many lymphomas exhibit resistance to cell death, suggesting a defective Fas signaling, which remains poorly understood. We previously reported that cells expressing the K1 protein of human herpesvirus 8 (HHV-8) resist death through the complex formation of the Ig-like domain of K1 with Fas. Recently, we investigated whether peptides derived from the Ig-like domain of the K1 protein may affect cell death.

**Methods:**

K1 positive and negative cell lines were incubated with the K1-derived peptides, and cell death (apoptotic and necrotic) was assessed by flow cytometry and LDH assay. Activation of caspases was assessed by fluorometric assay and flow cytometry. Fas receptor-independent, peptide-mediated cell killing was tested in the Fas-resistant Daudi cell line and Jurkat cell clones deficient in caspase-8 and FADD functionality. Activation of TNF receptors I and II was blocked by pre-incubation with corresponding blocking antibodies. The effect of the K1 peptide *in vivo* was tested in a mouse xenograft model.

**Results:**

We observed that the peptide S20-3 enhanced cell death in K1-positive BJAB cells and HHV-8 positive primary effusion lymphoma (PEL) cell lines. Similar effects of this peptide were observed in B-cell lymphoma and T-lymphoblastic leukemia cells without K1 expression but not in normal human peripheral blood mononuclear cells. A single intratumoral injection of the S20-3 peptide decreased the growth of Jurkat xenografts in SCID mice. The mechanism of tumor cell death induced by the S20-3 peptide was associated with activation of caspases, but this activity was only partially inhibited by the pan-caspase inhibitor z-VAD. Furthermore, the K1 peptide also killed Fas-resistant Daudi cells, and this killing effect was inhibited by pre-incubation of cells with antibodies blocking TNFRI.

**Conclusion:**

Taken together, these findings indicate that the S20-3 peptide can selectively induce the death of malignant hematological cell lines by Fas- and/or TNFRI-dependent mechanisms, suggesting the K1-derived peptide or peptidomimetic may have promising therapeutic potential for the treatment of hematological cancers.

## Background

The key to effective chemotherapy responses in cancer is the presence of the Fas receptor (CD95, Apo-1), a member of the tumor necrosis factor superfamily of cell death receptors [1]. These receptors form trimers in the plasma membrane and, upon the binding of their respective ligands, activate the initiator caspase-8 through the recruitment of adaptor proteins (FADD and/or TRADD) to the receptors’ death domains. In type I apoptosis, the activated caspase-8 directly activates executioner caspases. In type II apoptosis, caspase-8 cleaves Bid triggering permeabilization of the mitochondrial outer membrane, cytochrome C release, and propagation of the apoptotic signal downstream of the cascade [1].

Many studies suggest that drug-induced apoptosis occurs through Fas signaling; thus, defective Fas signaling could be responsible for the resistance to chemotherapy that is frequently observed in cancers [2-5]. Several studies have shown that the Fas-mediated cell-death pathway is altered in malignant hematological cells [6,7], which can be viewed as one of the mechanisms of resistance to chemotherapy. The CD44 isoforms v6 and v9, hepatocyte growth factor receptor/Met (HGFR/Met), and HHV-8 oncoprotein K1 have been shown to bind to Fas and regulate its activity [8-11]. Therefore, treatments targeting these Fas regulators in cancer cells could be an effective strategy to increase sensitivity to Fas-mediated apoptosis and to chemotherapy.

Lymphomas occur frequently in association with infectious agents such as the Epstein-Barr virus, human immunodeficiency virus, or HHV-8 [12,13]. We have shown that the HHV-8-derived K1 protein interacts with Fas and blocks apoptosis [8,10]. In the current study, we investigated whether peptides derived from the Ig-like domain of the K1 protein could alter K1-Fas interaction and, consequently, apoptosis in lymphoma cells. For this purpose, we treated K1-expressing cells as well as B-cell lymphoma and T-lymphoblastic leukemia cells with peptides corresponding to the Ig-like domain of K1, followed by cell death analysis. Our results show that the K1-derived S20-3 peptide kills lymphoma and leukemia cells *in vitro* and *in vivo* by a mechanism dependent on Fas and/or TNF-α receptors.

## Methods

### Cells

Human lymphoblastoma cell lines BJAB, Daudi; HHV-8-positive primary effusion lymphoma-derived B-cell lines BC-3, BCBL-1, KS-1; human T-lymphoblastic cell line Jurkat (all from ATCC, Manassas, VA), a caspase-8– and FADD–deficient Jurkat cell lines (I9.2 and I2.1) (donated by Dr. J. Chandra, The University of Texas MD Anderson Cancer Center) were grown in RPMI 1640 medium supplemented with 10% FBS (both from Mediatech, Herndon, VA) and maintained in a 5% CO_2_ atmosphere at 37°C. The 293T cells (ATCC) were cultured in Dulbecco’s modified Eagle's medium (DMEM) (Mediatech) supplemented with 10% FBS. Collection of blood samples was in accordance with approved MD Anderson Cancer Center protocol. Peripheral blood mononuclear cells (PBMCs) from healthy volunteers were isolated from heparinized venous blood by density gradient centrifugation and used immediately in the experiments. BJAB cells stably expressing K1 (BJABK1) were described previously [8,10].

### Peptide synthesis

Peptides were chemically synthesized by multiple peptide solid-phase synthesis (New England Peptide, Gardner, MA, and Celtek Bioscience, Nashville, TN). All peptides were purified to >95% purity by high-performance liquid chromatography. Peptide stocks (10 mM) were prepared in dimethyl sulfoxide (DMSO) (Thermo Fisher, Waltham, MA), and aliquots were stored at −20°C.

### Apoptosis analysis

Apoptosis analysis was performed using the FITC AnnexinV Apoptosis Detection Kit I, according to the manufacturer’s protocol (BD Biosciences, San Jose, CA). Cells were re-suspended in serum-free medium (1 × 10^6^/mL) and treated with either 100 μM peptide alone or combined with 20 μM z-VAD (BD Biosciences) for 1 hour, or pretreated for 15 minutes with the peptide and combined with 200 ng/mL of recombinant soluble Fas ligand (FasL) (Alexis, San Diego, CA) for the indicated times. Subsequently, cells were washed, re-suspended in a binding buffer containing AnnexinV-FITC and propidium iodide (PI), and analyzed by flow cytometry (FACSCalibur; Beckman-Coulter, Brea, CA) after 15 minutes of incubation.

### Caspase activity assay

The activities of caspase-8, -9, and -3 were determined by flow cytometry using the CaspGLOW^TM^ Fluorescein Active Caspase Staining Kit (BioVision, Mountain View, CA), according to the specifications of the manufacturer. Briefly, 1 × 10^6^ cells were seeded in serum-free medium and treated with 100 μM S20-3 peptide for 1 hour. Cells were then washed, cultured in medium containing 10% FBS for 3 hours, and, subsequently, incubated with 1 μl of FITC-IETD-FMK (for caspase-8 activity), FITC-LEHD-FMK (for caspase-9 activity), or FITC-DEVD-FMK (for caspase-3 activity) for 60 minutes at 37°C. Cells were washed twice and analyzed by flow cytometry.

### Immunoblotting

The cells (10 × 10^6^) were resuspended in 1 mL of lysis buffer (Cell Signaling Technology, Beverly, MA) supplemented with protease inhibitors (Roche), and incubated 1 hour on ice. One hundred micrograms of each extract were separated on 10% SDS-polyacrylamide gels (Bio-Rad Laboratories, Hercules, CA) and transferred to nitrocellulose membranes (Whatman Schleicher & Schuell, Keene, NH). Membranes were blocked at room temperature for 1 hour in blocking buffer (5% nonfat dry milk, 0.1% Tween-20 in PBS). Separated proteins were analyzed by Western blot with anti-GAPDH (1:1000, Santa Cruz Biotechnology, Santa Cruz, CA; loading control), anti-TNFRI and anti-TNFRII antibodies (1:1000, both kind gifts from Dr. B. B. Aggarwal, MD Anderson Cancer Center) overnight at 4°C. Blots were washed and then incubated with either anti-mouse (Santa Cruz Biotechnology) or anti-rabbit (Cell Signaling Technology) horseradish peroxidase-conjugated antibody (1:5000). The signal was visualized by chemiluminescence Western blot kit (PerkinElmer, Waltham, MA) and exposure to film (Amersham, Piscataway, NJ).

### LDH assay

Cells (1 × 10^6^) were pre-incubated for 1 hour with 5 μg/mL of TNFRI or TNFRII blocking antibodies (both from R&D Systems, Minneapolis, MN) at 37°C and then treated with TNF-α (10 ng/mL) (Life Technologies - Gibco, Carlsbad, CA) or the peptide S20-3 (100 μM) for 1 hour. After treatment, the growth medium was removed and stored at −20°C. An LDH assay was performed according to the manufacturer's protocol (BioVision). Standard media were used as blank controls; “high control” corresponds to the sample of cells treated with lysis solution. Results were normalized to control cells, and the percentage of necrotic cells was calculated using the following formula: % cytotoxicity = [(treated– control)/(high control– control)] × 100.

### *In vivo* tumor growth assay

All animal studies were conducted according to protocols approved by MD Anderson Cancer Center’s Institutional Animal Care and Use Committee. Jurkat cells (5 × 10^6^ per injection) were re-suspended in sterile PBS and subcutaneously injected into the right flank of 5-week-old CB17/SCID mice (Harlan Laboratories, Indianapolis, IN). When xenograft tumors reached 100 mm^3^, the mice were given a single intratumoral injection of peptides (33.9 mg/kg): S20-3, TCR, or vehicle; 4 mice each. The mice were killed 8 days after injection, and the tumor tissue was harvested. Tumor width (W) and length (L) were measured by calipers, and size was calculated using the formula W^2^× L/2. The tumoricidal activity was evaluated by comparison of tumor size among groups.

### Statistical analysis

The 2-tailed Student’s *t* test was used to estimate the statistical significance of the differences between results from triplicate samples or experiments, and the results are expressed as mean values ± standard deviations or standard errors, respectively. The level of significance was set at *P* < 0.05.

## Results

### S20-3 peptide induces cell death of BJABK1 cells

Our previous studies demonstrated that wild-type K1, but not a truncated K1 with the Ig-like domain deleted, binds to Fas and prevents Fas activation by FasL or by an agonistic Fas antibody [8,10]. To further elucidate K1-mediated regulation of Fas, we designed peptides derived from the Ig-like domain of K1 (Table 1), targeting the K1 binding site on the Fas receptor. 

**Table 1:** Protein sequence of the Ig-like domain of human herpesvirus 8 K1 protein and derived peptides

		
K1 Ig-domain		HSLWITWYPQPVLQTLCGQPSNTVTCGQYVTLYCSTSGNYVTVW
K1 peptides		
20 amino acids	S20-1	HSLWITWYPQPVLQTLCGQP (84–103)
	S20-2	PVLQTLCGQPSNTVTCGQYV (94–113)
	S20-3	SNTVTCGQYVTLYCSTSGNYV (104–124)
10 amino acids	S10-1	SNTVTCGQYV (104–113)
	S10-2	TVTCGQYVTL (106–115)
8 amino acids	S8-1	TVTCGQYV (106–113)
	S8-2	VTLYCSTS (113–120)

**Figure 1:**
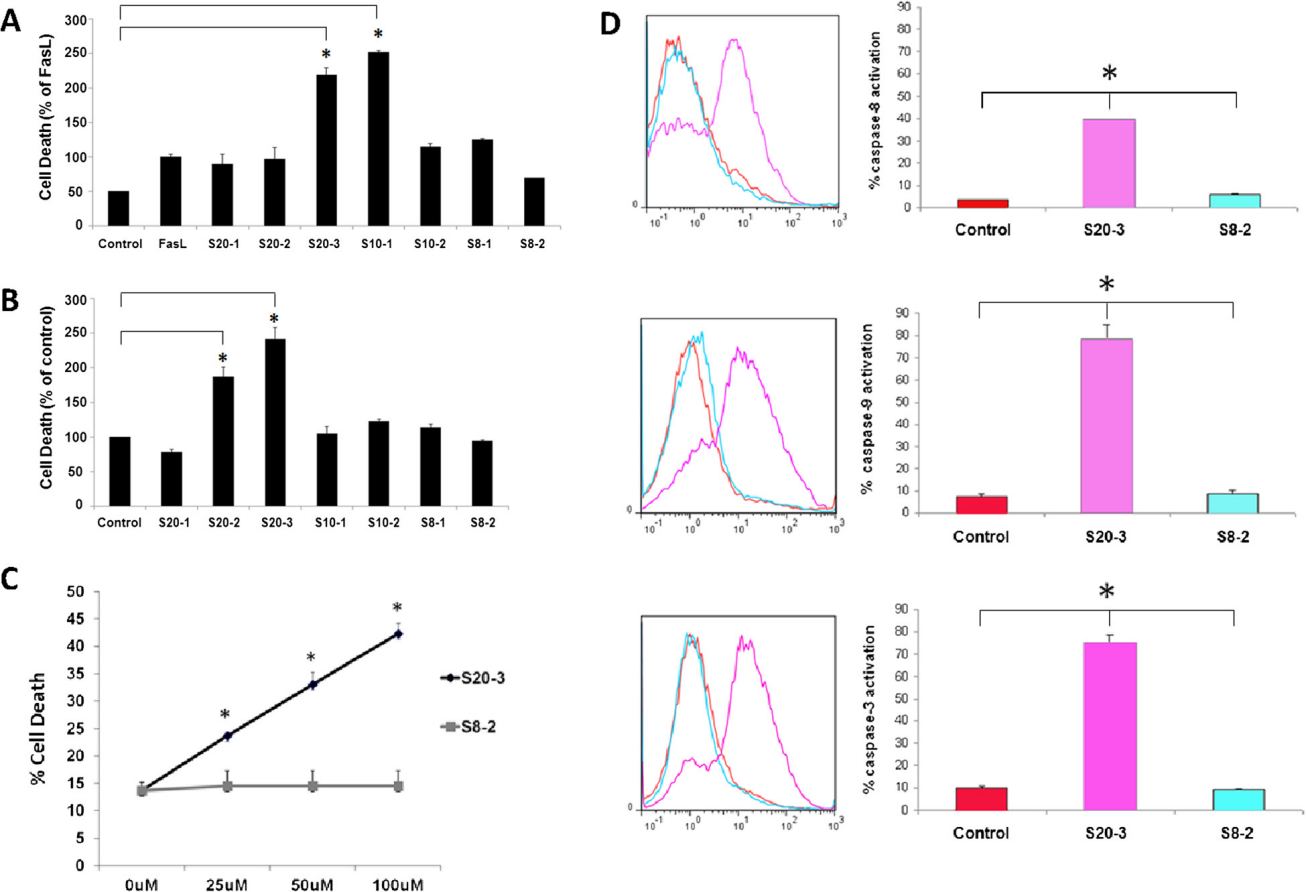
**A human herpesvirus 8 K1 peptide induces dose-dependent cell death and activates caspase cascade in BJABK1 cells.** BJABK1 cells were pulse-treated for 1 hour with 100 μM concentration of indicated Ig-like domain-derived peptides and 200 ng/mL of FasL (**A**), 100 μM concentration of the indicated peptide alone (**B**), and increasing concentrations of S20-3 or S8-2 peptides (**C**). Cells were, subsequently, incubated in a complete medium for 24 hours, stained with AnnexinV/PI, and examined by flow cytometry. (**D**) BJABK1 cells were treated with 100 μM peptide or DMSO for 1 hour. The cells were then washed and incubated in complete medium for 4 hours. Fluorometric caspase activity was analyzed by flow cytometry. The results are presented as means ± SD of triplicate wells. Asterisks indicate statistically significant differences compared with control treatment; *P < 0.05.

We first investigated whether K1 peptides could sensitize the Burkitt’s lymphoma cell line BJAB stably expressing K1 (BJABK1) to Fas-mediated apoptosis. Cells were treated with 100 μM peptide in combination with 200 ng/mL of FasL for 24 hours, followed by analysis of apoptosis by flow cytometry. The combination of S20-3 and S10-1 peptides with FasL showed a significant (2.2- and 2.5-fold, respectively) increase in cell death compared with FasL alone (Figure 1A). No significant differences in apoptosis rates were seen with FasL in combination with other K1-derived peptides shown in Table 1 (20–1, 20–2, S10-2, S8-1, S8-2).

In the control experiment, BJABK1 cells were treated with 100 μM peptides or buffer for 1 hour, and apoptosis was evaluated 24 hours after treatment by flow cytometry. Surprisingly, two of the longest overlapping peptides (S20-2 and S20-3) individually induced a significant (1.9- and 2.4-fold, respectively) increase in apoptotic cell death in the BJABK1 cells compared with buffer control (Figure 1B). None of the other peptides overlapping the 20-amino acid sequence of the peptide S20-3 (Table 1) showed a significant apoptotic effect.

The S20-3 peptide showed a reproducible, dose-dependent increase in apoptotic cell death (up to 40% at 100 μM) as early as 4 hours after treatment, while the control peptide S8-2 was ineffective at all tested concentrations (Figure 1C). Further studies were performed to understand the underlying mechanism for the induction of cell death by the S20-3 peptide.

The proper control for the peptide activity would have been a scrambled S20-3-derived peptide. However, we encountered difficulty obtaining reasonable quantities of any S20-3-derived scrambled peptide of desired purity (>95%), suitable for the experiments. One possibility was to use inactive 20-mer peptide S20-1 as a negative control, but this peptide does not share any residues with the active S20-3 peptide. Based on the results in Figure 1A and B, the S8-2 peptide, which overlaps part of S20-3 peptide, was included as negative control reagent in subsequent studies.

### The S20-3 peptide activates caspases and triggers apoptosis in BJABK1 cells

Stimulation of the Fas death receptor results in the recruitment of the adaptor protein, FADD, and activation of caspase-8, which initiates propagation of the death signal down the caspase cascade [14,15]. To determine the involvement of caspase-8, -9, and -3 in the cell death induced by the S20-3peptide, we used caspase-specific fluorescently-tagged substrates to monitor caspase activation. In the BJABK1 cells, exposure to S20-3 significantly (*P* < 0.01) increased the activity of all caspases tested: caspase-8 (39.6% vs. 3.7%), caspase-9 (78.3% vs. 7.4%) and caspase-3 (75.2% vs. 10.2%) (Figure 1D). These findings indicate the role of the caspase-8–initiated apoptotic pathway in S20-3 peptide-induced cell death. The control S8-2 peptide showed no effect on caspases’ activity (Figure 1D).

Another important feature of apoptosis is a decrease of the mitochondrial membrane potential (Ψm) [16]. Changes in the Ψm in cells exposed to peptides S20-3 and S8-2, or the agonistic Fas antibody CH-11 as positive control, were measured by staining with tetramethylrhodamine ethyl ester (TMRE). A decreased TMRE signal corresponding to decreased membrane potential was observed in a significant number of S20-3 peptide-treated (20%) and CH-11–treated (22%) cells as early as 4 hours after treatment, relative to treatment with buffer or the control S8-2 peptide (Additional file 1: Figure S1).

### The S20-3 peptide is effective against various hematological cancer cell lines

**Figure 2:**
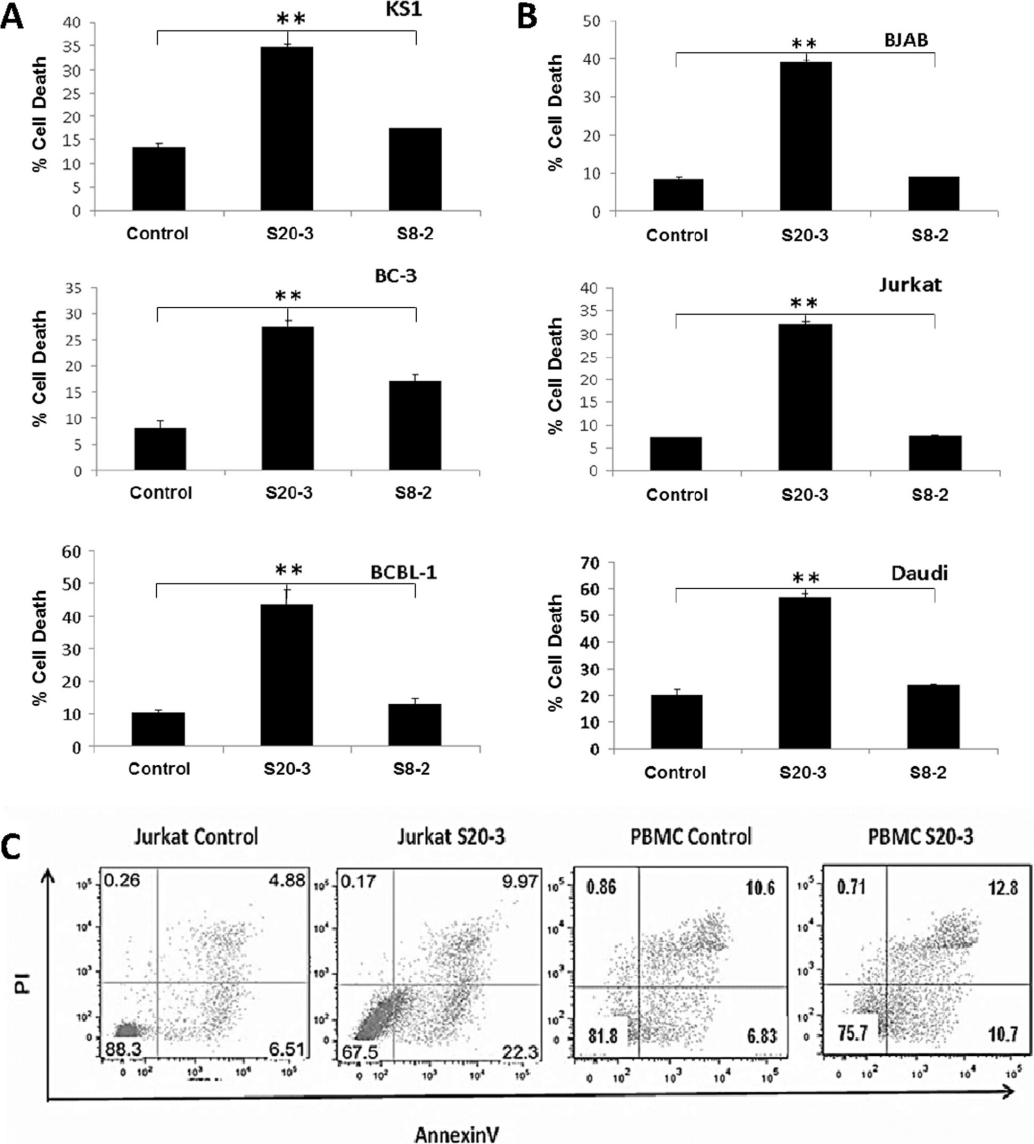
**The HHV-8 K1-derived peptide S20-3 induces cell death in K1-positive and K1-negative hematological cancer cells but not in PBMCs from healthy donors.** Indicated cell lines (1 × 10^6^ cells/mL) were incubated with 100 μM peptide S20-3 or buffer for 1 hour. Cells were washed and incubated in complete medium for 24 hours before flow cytometry analysis. (**A**) HHV-8– and K1-positive cell lines KS-1, BC-3, BCBL-1; (**B**) HHV-8 and K1-negative cell lines BJAB, Jurkat, Daudi; (**C**) Jurkat cells and PBMCs from healthy donors. Data in (A) and (B) are shown as the means ± SD of triplicate wells. Double asterisks indicate significant differences compared with control treatments; **P < 0.01. Panel (**C**) shows representative results of 2 experiments with samples analyzed in triplicates.

We further investigated whether the S20-3 peptide would be effective in inducing cell death in HHV-8–positive cancer cell lines (KS-1, BC-3, BCBL-1), which have been shown to express K1 [10]. All HHV-8–infected cell lines tested were sensitive to the S20-3 peptide, which induced death in about 20–35% of cells, whereas no significant effect on cell death was detected with the S8-2 control peptide (Figure 2A). 

To evaluate whether the peptides were able to modulate the interaction between Fas and K1, 293T cells were transiently transfected with the vector expressing Flag-tagged K1 protein, lysed, and subjected to co-immunoprecipitation analysis used previously to show a direct physical interaction of Fas with K1 [8]. We observed that K1-Fas interaction was not disrupted by incubation of cells with the S20-3 or other K1-derived peptides with the exception of the shorter peptide S10-1 (Additional file 1: Figure S2).

The lack of S20-3 peptide’s effect on the K1-Fas interaction suggested a possible cell-killing mechanism independent of K1. To confirm this hypothesis, we tested the peptide’s ability to kill K1-negative cell lines. The S20-3 peptide was able to induce significant levels of cell death in K1-negative BJAB cells (30%) and in the T-cell leukemia Jurkat cell line (25%) (Figure 2B). Quite surprisingly, the S20-3 peptide was equally effective in killing Daudi cells (35%), which express low levels of Fas on the cell surface and are considered Fas-resistant [17].

In contrast, human PBMCs from healthy donors, treated with S20-3 peptide, showed no significant amount of cell death (Figure 2C). Overall, S20-3 peptide treatment induced a 4.6 ± 1.6% increase in cell death in 3 PBMC control samples from different donors, whereas the same treatment induced a 23.8 ± 5.7% increase in death of Jurkat cells. These results suggest that the S20-3 peptide derived from the HHV-8 K1 protein selectively induces cell death in malignant hematological cells, but is not toxic to normal human cells.

### The S20-3 peptide kills cells in the absence of the Fas receptor

**Figure 3:**
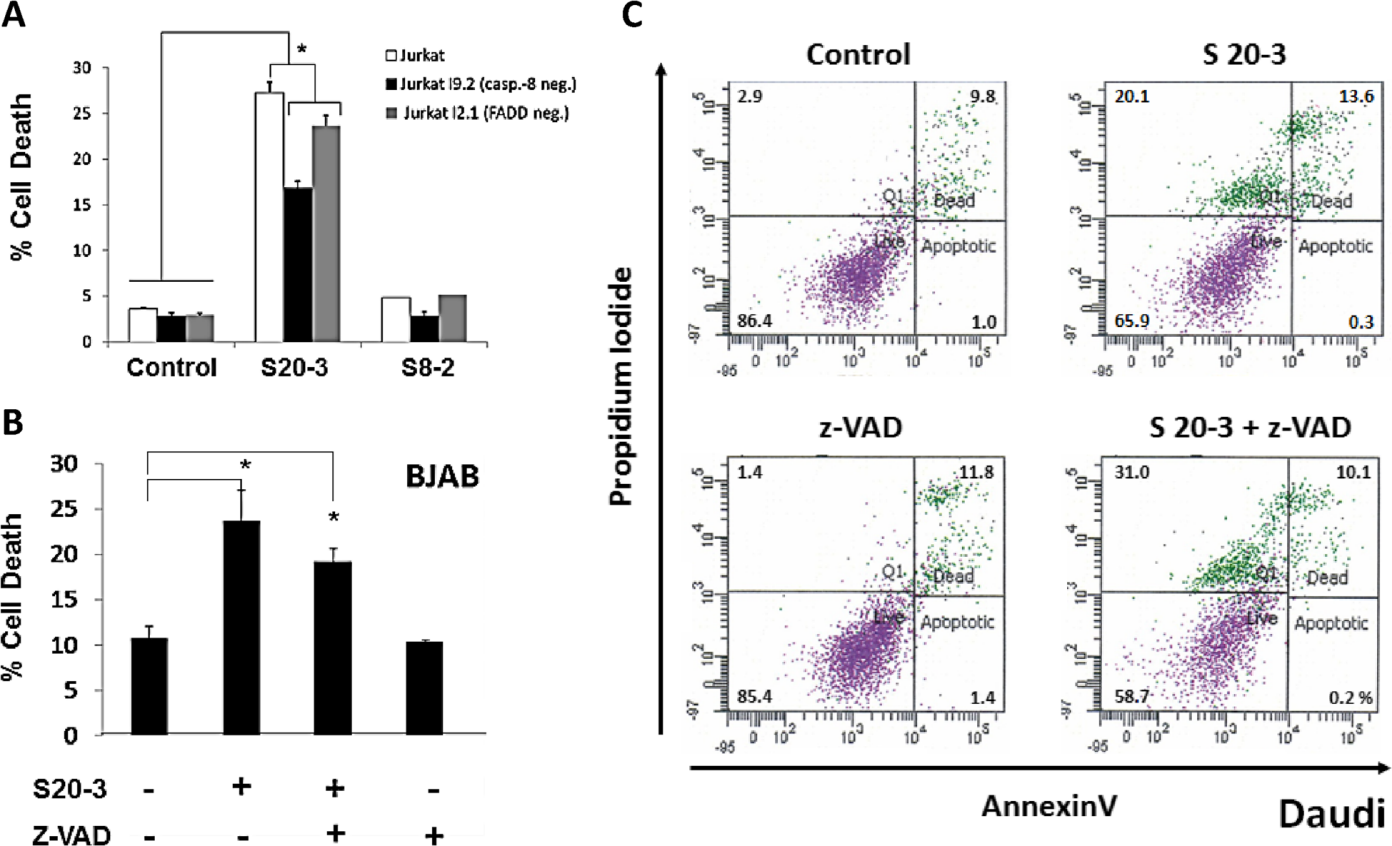
**The S20-3 peptide–induced cell death is only partially dependent on caspases and involves necroptosis.** (**A**) Jurkat (wild-type), Jurkat I9.2 (caspase-8–deficient), and Jurkat I2.1 (FADD-dominant-negative mutant) cell lines were incubated with 100 μM peptide S20-3. (**B**) BJAB cells were incubated with 100 μM peptide S20-3 in the presence or absence of 20 μM pan-caspase inhibitor z-VAD-FMK. (**C**) Daudi cells were incubated with 100 μM peptide S20-3 or buffer in the presence or absence of 20 μM pan-caspase inhibitor z-VAD-FMK. After 1 hour of incubation, cells were washed and incubated in complete medium for 24 hours before flow cytometry analysis. Data in (A) and (B) are shown as means ± SD of triplicate wells; *P < 0.01.

To investigate whether S20-3–induced apoptosis depends on the signaling of the Fas receptor, we tested the S20-3 peptide in Fas-resistant Jurkat cell lines I2.1 and I9.2, which have defective FADD and caspase-8 functions, respectively [18]. The S20-3 peptide induced slightly less cell death in I2.1 cells than in the wild type Jurkat cells (21% vs. 24% above control; Figure 3A). The response of caspase-8 function-defective Jurkat cell line I9.2 to the S20-3 peptide was significantly blunted compared with that of wild-type Jurkat cells (14.4% vs. 24% above control; 60% reduction) but not completely eliminated (Figure 3A). In line with this result, we found that the pan-caspase inhibitor z-VAD also only partially blocked S20-3-induced death in BJAB cells (8.9% vs. 13.3% above control; 67% reduction) (Figure 3B) as well as apoptosis induced by the Fas-agonistic antibody CH-11 (14% vs. 29% above control; 48% reduction) (data not shown). 

Further examination of the cell death induced by the S20-3 peptide in Daudi cells revealed that the S20-3 peptide induced necrosis (33.7% PI–positive cells) rather than apoptosis (0.3% AnnexinV–positive/PI–negative cells) in Fas-resistant Daudi cells (Figure 3C and Additional file 1: Figure S3A), and z-VAD further enhanced this effect (41.1% PI–positive cells) (Figure 3C). An LDH release assay further confirmed that the S20-3 peptide was causing necrosis as early as 1 hour post treatment (Additional file 1: Figure S3B).

### Cell killing by the S20-3 peptide involves TNF-α receptors

The fact that the S20-3 peptide induced necrosis of Fas-resistant Daudi cells, together with the observed rapid kinetics of cell killing (significant cell death at 4 hours), pointed to the possibility that the S20-3 peptide is promiscuous and interacts not only with Fas, but it may also engage one of the closely related death receptors sharing a significant structural similarity [19]. Such promiscuity is not unprecedented. For example, IFN-α–treated Daudi cells upregulate expression of TNF-α and Fas. Produced TNF-α then activates the closely related Fas receptor [20]. Based on these facts, we hypothesized that a peptide designed to bind Fas receptor may also interact with and affect the TNF receptor.

**Figure 4:**
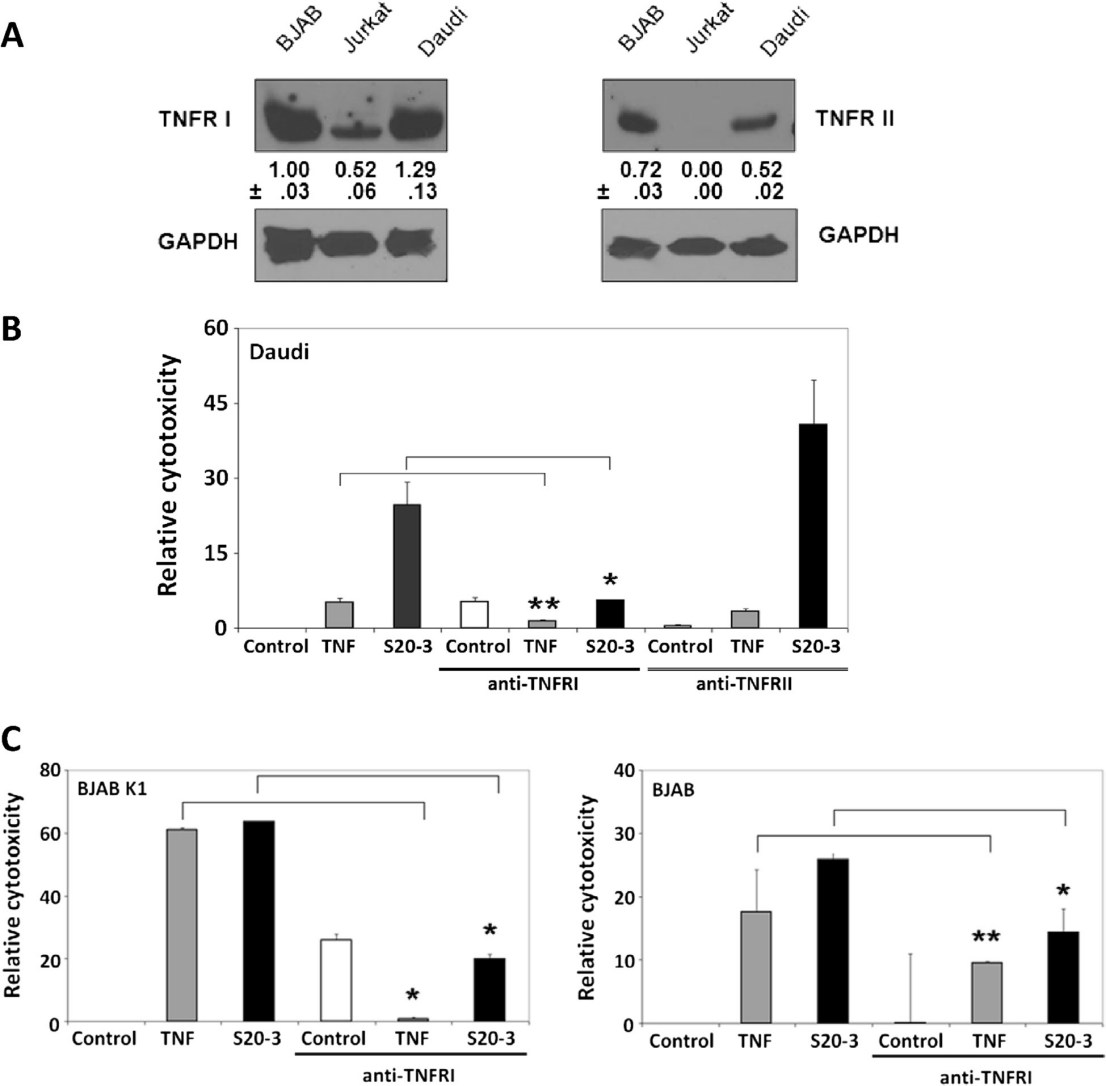
**The S20-3 peptide–induced cell death involves TNFRI.** (**A**) Immunoblot analysis of total cellular levels of TNF receptors I and II in BJAB, Jurkat, and Daudi cells. Numbers represent expression levels relative to GAPDH (loading control). (**B**) Daudi cells were pre-incubated for 1 hour with 5 μg/mL of TNFRI- or TNFRII-blocking antibodies, followed by 1 hour of treatment with 5 ng/mL of TNF-α or 100 μM peptide S20-3, and immediately analyzed for necrosis by LDH release assay. (**C**) BJABK1 cells (left panel) and BJAB cells (right panel) were pre-incubated for 1 hour with 5 μg/mL of TNFRI-blocking antibody, subsequently treated with 100 μM peptide S20-3 or 5 ng/mL of TNF-α for 1 hour, and analyzed as in (**B**). The relative cytotoxicity values in (**B**) and (**C**) were calculated as LDH release in [(treated-control)/(high control-control)]x100 and are shown as means ± SD of triplicate wells; *P < 0.005, **P < 0.02.

We first evaluated the expression levels of TNFRI and TNFRII in BJAB, Jurkat, and Daudi cells and found that all 3 cell lines expressed TNFRI, but only BJAB and Daudi cells expressed detectable levels of TNFRII (Figure 4A). We next evaluated the effect of TNFR-blocking antibodies on necrosis induced by TNF-α or S20-3 peptide by measuring LDH release as early as 1 hour after treatment to evaluate necrosis/necroptosis rather than post-apoptotic secondary necrosis [21]. Figure 4B clearly shows that pre-incubation of Daudi cells with the TNFRI blocking antibody decreased TNF-α and S20-3 peptide induced necrosis/necroptosis, while the TNFRII-blocking antibody showed a rather enhanced killing. The latter finding is consistent with the inhibition of pro-survival signaling mediated by TNFRII [22] by the blocking antibody. These results suggest that, besides Fas, TNFRI is also targeted by S20-3. We then tested the effect of TNFRI-blocking antibody on peptide-induced necroptosis in TNFRI-positive BJABK1 and BJAB cells. In both cell lines, the TNFRI-blocking antibody significantly decreased death induced by TNF-α and S20-3 peptide (Figure 4C). However, the TNFRI-blocking antibody-mediated inhibition of cell-killing was more prominent in BJABK1 cells, where the S20-3 peptide binding to Fas is blocked by K1 (a lack of displacement of K1 from Fas by S20-3 peptide; Additional file 1: Figure S2). Thus, in this case, the peptide acts primarily on TNFRI. On the other hand, TNFRI-blocking antibody affected cytotoxicity of TNF-α and S20-3 peptide to a lesser extent in BJAB cells, consistent with the availability of Fas for peptide S20-3 binding in the absence of K1 and, thus, for primary peptide signaling effects. 

**Figure 5:**
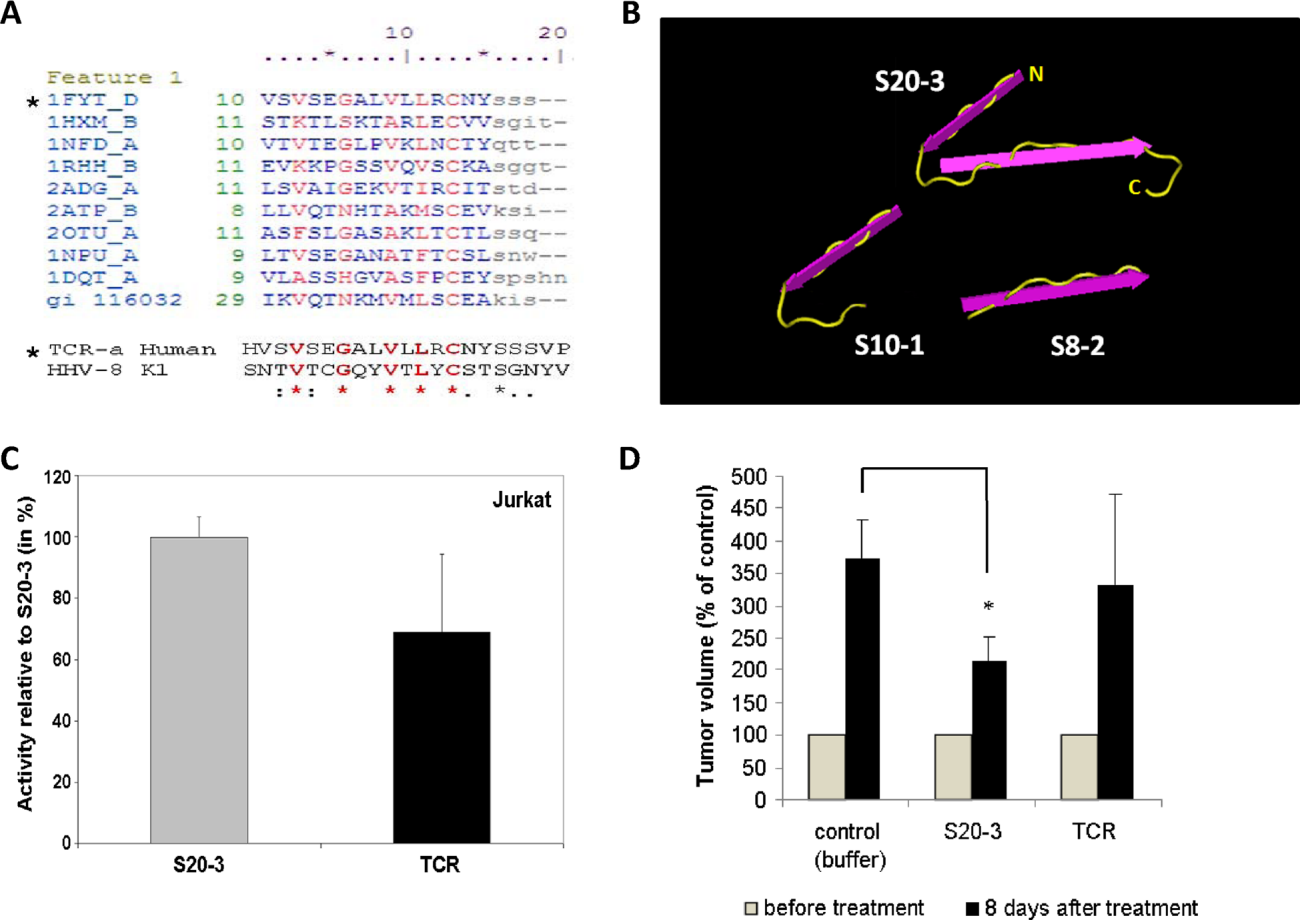
**The S20-3 peptide, but not the structurally similar TCR-derived peptide, significantly suppresses growth of Jurkat cell xenografts.** (**A**) Sequence alignment of the relevant regions of the Ig-V domains based on the known structures (http://www.ncbi.nlm.nih.gov/Structure/cdd/cddsrv.cgi?hslf=1&uid=cd00099&#seqhrch) and the sequence comparison of S20-3 with the corresponding human TCR-α-derived peptide. (**B**) Predicted structures of S20-3, S10-2, and S8-2 peptides extracted from the structure of TCR-α (Protein Database ID 1FYT) using Cn3D 4.3 software (www.ncbi.nlm.nih.gov/Structure/CN3D/cn3d.shtml*)*. (**C**) Jurkat cells were treated with 100 μM peptides (S20-3, TCR) or buffer for 1 hour and, subsequently, incubated in complete medium for 24 hours. Cell killing was analyzed by flow cytometry, and background death (buffer) was subtracted. Values are presented as the means of the percentage of activity relative to the activity of S20-3 ± SE from 3 independent experiments. (**D**) Flanks of SCID mice were injected with 5 × 10^6^ Jurkat cells. Two weeks later, tumors were injected with a single dose of S20-3, TCR peptide, or vehicle (DMSO) in 50 μL of saline (4 mice each). Eight days after treatment, mice were killed and the tumors were harvested and measured. Tumor measurements are reported as means ± SD; *P < 0.05.

The S20-3 peptide corresponds to the Ig-like domain of K1 and shares the conserved residues with other Ig-like domains (Figure 5A). To further explore structure-related promiscuity, we tested a 20–amino acid peptide derived from the Ig-like domain of the human T-cell receptor (TCR) (Figure 5A), homologous to the peptide S20-3 from K1. Both peptides share 5amino acid residues common to the Ig-like domains and exhibit high hydrophobicity. The TCR peptide showed 60–80% of the cell death-inducing activity of the S20-3 peptide in 3 independent experiments (Figure 5C), further underscoring a mechanism involving possible structural promiscuity of peptides and/or receptors.

### Inhibition of tumor growth by the S20-3 peptide in a xenograft model

The SCID mice injected subcutaneously with Jurkat cells developed solid tumors at the inoculation site. Using this model, we tested the ability of the peptide S20-3 to alter growth of xenograft tumors*.* Mice received a single intratumoral injection of vehicle, S20-3, or TCR peptide. Treatment with the S20-3 peptide resulted in a modest but significant (*P* < 0.05) suppression of tumor growth 8 days after injection compared with vehicle control (Figure 5D). In line with our *in vitro* results, the TCR peptide showed a smaller suppressive effect on tumor growth, without statistical significance. Importantly, the mice treated with the peptides did not exhibit signs of toxicity, such as agitation or impaired movement and posture. These results support intratumoral administration of the S20-3 peptide as a potentially safe approach for inducing significant inhibition of xenograft tumor growth.

## Discussion

In this report, we present evidence showing that the peptide S20-3, corresponding to the Ig-like domain of the Fas-targeting K1 protein of HHV-8, selectively kills hematological cancer cells, and the mechanism involves the Fas and TNFRI receptors. The cell-killing effect appears to be selective for cancer cells *in vitro. In vivo,* even a single intratumoral dose of peptide was active against the growth of xenograft tumors.

From the array of K1 Ig-like domain peptides tested (Table 1), only the S20-3 peptide demonstrated strong and reproducible cell-killing activity (Figure 1 and Figure 2) in all 6 hematological cell lines tested but not in PBMC controls (Figure 2). While it is not clear as to why S20-3, and also less reproducibly S20-2, but not other K1 Ig-like domain-derived peptides, possess cell-killing activity, the structural features of the predicted Ig-domain (Figure 5B) reveal a unique feature of the S20-3 peptide; a loop (centered at conserved glycine residue) linking 2 beta sheets, which are predicted to be destabilized or absent in the rest of peptides tested (Table 1). A truncated version of the S20-3 peptide, S10-1, representing the first beta sheet and the loop (Figure 5B), as well as S8-2 peptide, representing the second beta sheet (Figure 5B), lack cell killing properties (Figure 1B). On the other hand, a TCR-derived peptide sharing 5 structure-defining residues with S20-3 (Figure 5A) also showed cell-killing effect (Figure 5C), suggesting that the biological effect of S20-3 is related to its structure.

A seemingly contradictory effect of the whole Ig-like domain in K1 protein and S20-3 peptide on Fas signaling may also be explained by the structure-function relationship. The fact that peptide S10-1, but not S20-3 or any other K1 peptide, was able to disrupt the K1-Fas complex (Additional file 1: Figure S2) suggests that first beta sheet is involved in K1-Fas interaction. This is further supported by the fact that peptide S10-2, lacking 3 residues from the first beta sheet, failed to displace K1 (Additional file 1: Figure S2) and did not show any enhancement of FasL activity (Figure 1A). Additionally, peptide S20-2, which also contains S10-1 residues, showed cell-killing properties similar to peptide S20-3, but with reduced reproducibility, suggesting that the second beta sheet in peptide S20-3 increases structural stability of the peptide and the additional residues, preceding (S20-2) or following (S20-3) S10-1 region, affect peptide behavior. Taking all this into account, we hypothesize that the smaller size and possible flexibility of the loop within S10-1peptide as compared to S20-3 peptide (Figure 5B) allow access of this peptide to the K1 binding site and, thus, displacement of K1 from Fas (Additional file 1: Figure S2). The second beta sheet in the S20-3 peptide stabilizes the loop, but at the same time, it decreases loop flexibility and increases bulkiness of the peptide, limiting its access to the K1 binding site in the presence of the K1 protein.

This hypothesis also helps explain the differential effects of the K1 Ig-like domain, S10-1, and S20-3 on Fas receptor activation. The S10-1 sequence within the Ig-like domain in the whole K1 protein is flanked by additional domains of K1 protein. Assuming the S10-1 region within K1 is exposed and available to bind Fas, the limitations of the movement imposed by surrounding K1 domains “lock” the Fas receptor in the closed conformation, preventing binding of FasL described previously [8]. On the other hand, the beta sheet and flexible loop in the S10-1 peptide can also bind the receptor, but without the rigidity of surrounding structures, its binding does not affect receptor conformation. Therefore, the S10-1 peptide has no direct effect on the receptor on its own, but sensitizes K1-positive cells to FasL (Figure 1A) by displacing the K1 protein (Additional file 1: Figure S2). The S20-3 peptide, more rigid and bulkier that S10-1peptide, can bind Fas only in the absence of K1. Without the flanking domains of the K1 protein and the whole Ig-like domain, S20-3 (and S20-2) can bind Fas receptor similarly to S10-1, but the presence of additional residues/structures induces conformational change mimicking the active state of the receptor.

The extrinsic apoptotic pathway involves activation of death receptors, recruitment of FADD, cleavage of pro-caspase-8, activation of caspases' cascade, and a drop in mitochondrial membrane potential [1]. While the precise target for the cell-killing activity of the S20-3 peptide is unclear, data presented here clearly show that the peptide activates caspase-8, -9, and -3 (Figure 1D) and decreases mitochondrial membrane potential (Additional file 1: Figure S1), suggesting involvement of a death receptor, such as Fas. However, a conventional dose of the pancaspase inhibitor z-VAD blocked cell killing only incompletely (Figure 3B), and Jurkat cells with mutated inactive caspase-8 or dominant-negative FADD also showed only partial blockage of S20-3–induced cell-killing (Figure 3A), despite their inability to form the death-inducing signaling complex (DISC) [23]. This persistence of the S20-3 peptide to kill mutant Jurkat cells (Figure 3A), the killing of Daudi cells that are considered Fas-resistant [17,24], the increase of necrotic death in the z-VAD-treated Daudi cells (Figure 3C and Additional file 1: Figure S3A), and their relatively fast killing [necrotic cell death in Daudi cells was detectable 1 hour after peptide exposure (Additional file 1: Figure S3)] suggested to us that S20-3 also activates a TNF receptor.

Even though Fas belongs to the TNF receptor family and shares a significant structural similarity with TNFR [19], the outcomes of activating these receptors can be quite different [25]. For example, activation of Fas receptor in L929 cells triggers apoptosis, whereas activation of TNFR triggers necrosis [26]. Owing to the structural similarity, TNF-α is able to also bind and activate the Fas receptor [20]. We, thus, investigated the possibility that, because of the structural promiscuity (further supported by the killing properties of a structurally related TCR peptide), the S20-3 peptide designed to bind the Fas receptor may also bind TNFR and trigger necrosis. We detected TNFRI expression in BJAB, Jurkat, and Daudi cells (Figure 3), and the TNFRI-blocking antibody significantly inhibited S20-3– and TNF-α–induced cell killing in all 3 cell lines (Figure 4B and C). On the contrary, the TNFRII-blocking antibody showed no inhibitory effect on the S20-3 cell-killing of TNFRII-positive Daudi cells (Figure 4B). This finding is not surprising considering the fact that activation of TNFRII triggers pro-survival signaling in hematological cancer cells [22], and activation of TNFRI is required for any death signaling from TNFRII due to the lack of a death domain in TNFRII [27].

 Our results with FADD– and caspase-8–defective Jurkat cells are in agreement with the reports showing that under apoptosis-deficient conditions (such as non-functional caspase-8 or FADD), stimulation with FasL or TNF-α could induce cell death with morphological features of necrosis/necroptosis [21,28,29]. Furthermore, lack of FADD, but not of caspase-8, was shown to sensitize Jurkat cells to TNF-α–induced necrosis [30]. Smac mimetic BV6 enhanced TNF-induced cell death in leukemia cells in 2 different ways: necroptosis, when the cells were apoptosis resistant (FADD– and caspase-8–deficient), and caspase-8–dependent apoptosis in apoptosis-proficient cells [31].

We hypothesize that the different death pathways can be activated in response to S20-3 treatment in Jurkat, Daudi, and BJAB cells, depending on the availability of and sensitivity to Fas and TNFRs. Another possibility is a cross talk between signaling events from TNF and Fas receptors, as reported by Takada et al., in which TNFRI is recruited by Fas to induce apoptosis [32].

An additional important observation is that the S20-3 peptide activity seemed to be specific to malignant cells; leukemia T cells displayed a much greater sensitivity to S20-3 than nonmalignant cells (Figure 2C). While the constitutive expression of TNF receptors was clearly demonstrated in most tumor cells, in normal peripheral lymphocytes, the expression of TNF receptors is subjected to a positive and negative regulation and can be induced by different stimuli [33,34]. However, normal unstimulated PBMCs express very low amounts of mRNAs for TNFRII > TNFRI > Fas [35], and normal lymphocytes were shown to be resistant to stimulation with activating antibodies targeting TNFRI, TNFRII, or Fas [36]. Thus, our findings of cancer-specific killing by the S20-3 peptide are in agreement with these reports.

## Conclusions

This is the first report where a peptide derived from the Ig-like domain of the virus-encoded protein effectively induces cell death, specifically in human lymphoma and leukemia cells with minimal toxicity to normal PBMCs and, thus, may expose a novel alternative to conventional chemotherapy, which may also be applied to other cancer types.

## Competing interests

The authors declare that they have no competing interests.

## Authors’ contributions

UD performed peptide experiments testing responses of Fas and wrote the manuscript; CK performed experiments testing responses of TNF receptors and wrote the manuscript; RHT, JW, XA performed mouse experiments and edited the manuscript; ZB and FS designed the experiments and wrote the manuscript. All authors read and approved the final manuscript.

## Supplementary Material

Additional file 1 Methods. Click here for file
