# Supplementary material for: The peptide derived from the Ig-like domain of human herpesvirus 8 K1 protein induces death in hematological cancer cells
Source: J Exp Clin Cancer Res. 2012 Aug 28;31(1):69. doi: 10.1186/1756-9966-31-69 (PMC3517441; doi:10.1186/1756-9966-31-69)
Supplement: Additional file 1 — Methods. [file 1756-9966-31-69-S1.pdf]

Additional files

Methods

Determination of mitochondrial membrane potential

The mitochondrial membrane depolarization (ΔΨm) was evaluated with tetramethylrhodamine ethyl ester (TMRE; Molecular Probes). Cells (1×10^6/mL) were treated as described in Materials and Methods and then incubated with 25 nM TMRE for 30 minutes at 37°C. After washing, cells were immediately analyzed by flow cytometry.

Transient Transfection and immunoprecipitation

The 293T cells were transfected with plasmid expressing Flag-tagged K1 protein using Lipofectamine 2000 (Gibco/Invitrogen) according to manufacturer's instructions. Twenty-four hours post transfection, cells were incubated for 1 hour with the indicated peptide (100 μM), or buffer control. Cells were subsequently lysed and aliquots removed for immunoblotting analysis of input material. Remaining supernatant was subjected to immunoprecipitation usig agarose-conjugated anti-Fas antibody B-10 (Santa Cruz Biotechnology), according to the manufacturer's instructions. Precipitated proteins and saved aliquots were combined with 60 μL of sample loading buffer (Cell Signaling Technology), and boiled for 5 minutes. Equal amounts were loaded onto 10% SDS gel. Separated total cellular proteins and precipitated proteins were visualized using HRP-conjugated anti-Flag (Sigma-Aldrich) and anti-Fas B-10 antibodies (Santa Cruz Biotechnology).

Figure Legends

Figure S1. The S20-3 peptide induces a decrease of mitochondrial membrane potential. Jurkat cells were treated with 100 μM peptide S20-3, DMSO (control), or 100 ng/mL of agonistic anti-Fas antibody CH-11 for 1 hour. The cells were then washed and incubated in complete medium for 4 hours. Changes in mitochondrial membrane potential ΔΨm were assessed by flow cytometry using the tetramethylrhodamine (TMRE) assay. The results are presented as means of percentages of cells with decreased potential ± SD of duplicate samples; $*P < 0.01$.

Figure S2. The effect of peptides on K1-Fas association

The 293T cells, transiently expressing Flag-tagged K1 protein, were incubated with 100 μM peptides for 1 hour. Cell lysates were analyzed for K1-Fas interaction by immunoprecipitation using anti-Fas antibody B-10, followed by immunoblotting using HRP-conjugated anti-Flag and anti-Fas antibodies. Numbers represent amounts of precipitated K1 protein relative to precipitated Fas.

Figure S3. The S20-3 peptide induces death of Daudi cells mainly by necrosis. Daudi cells were treated with 100 μM S20-3 peptide for 1 hour. **(A)** Cells were stained with AanexinV/PI and analyzed by flow cytometry. Average values ± SD from 3 independent experiments are presented. **(B)** Necrotic cell death was evaluated using the LDH assay according to the manufacturer's instructions (Biovision). Each bar represents an average value ± SE from 3 separate experiments.

Fig. S1

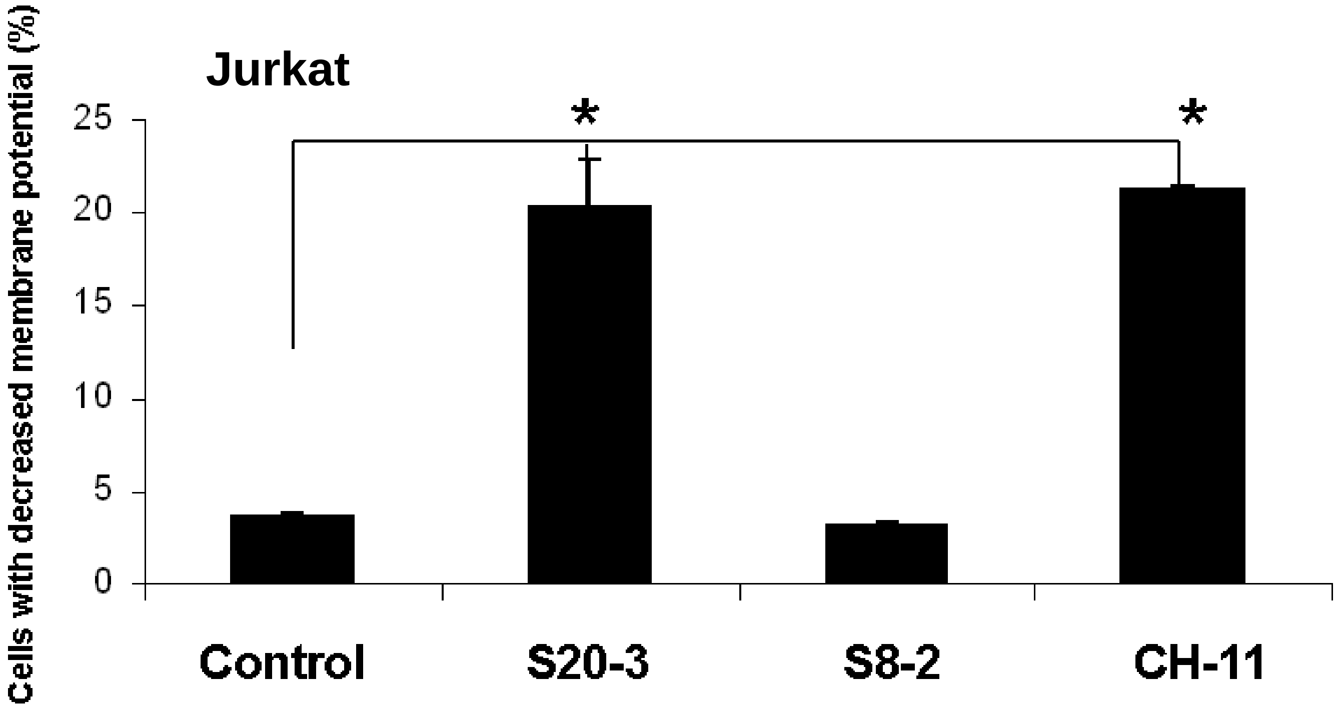
Jurkat
Cells with decreased membrane potential (%)
25
20
15
10
5
0
*
*
Control
S20-3
S8-2
CH-11

Fig. S2

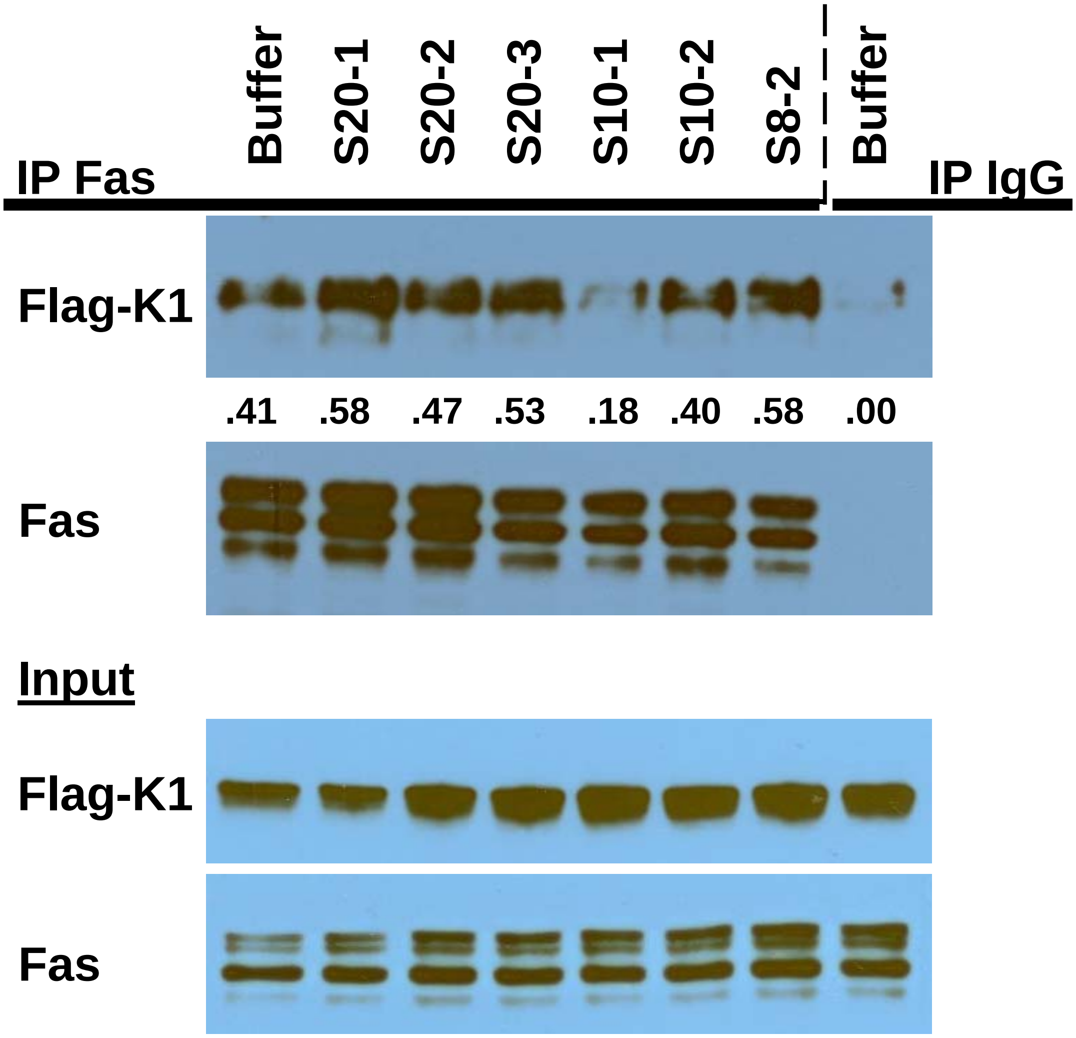
IP Fas
Buffer
S20-1
S20-2
S20-3
S10-1
S10-2
S8-2
Buffer
IP IgG
Flag-K1
.41
.58
.47
.53
.18
.40
.58
.00
Fas
Input
Flag-K1
Fas

Fig. S3

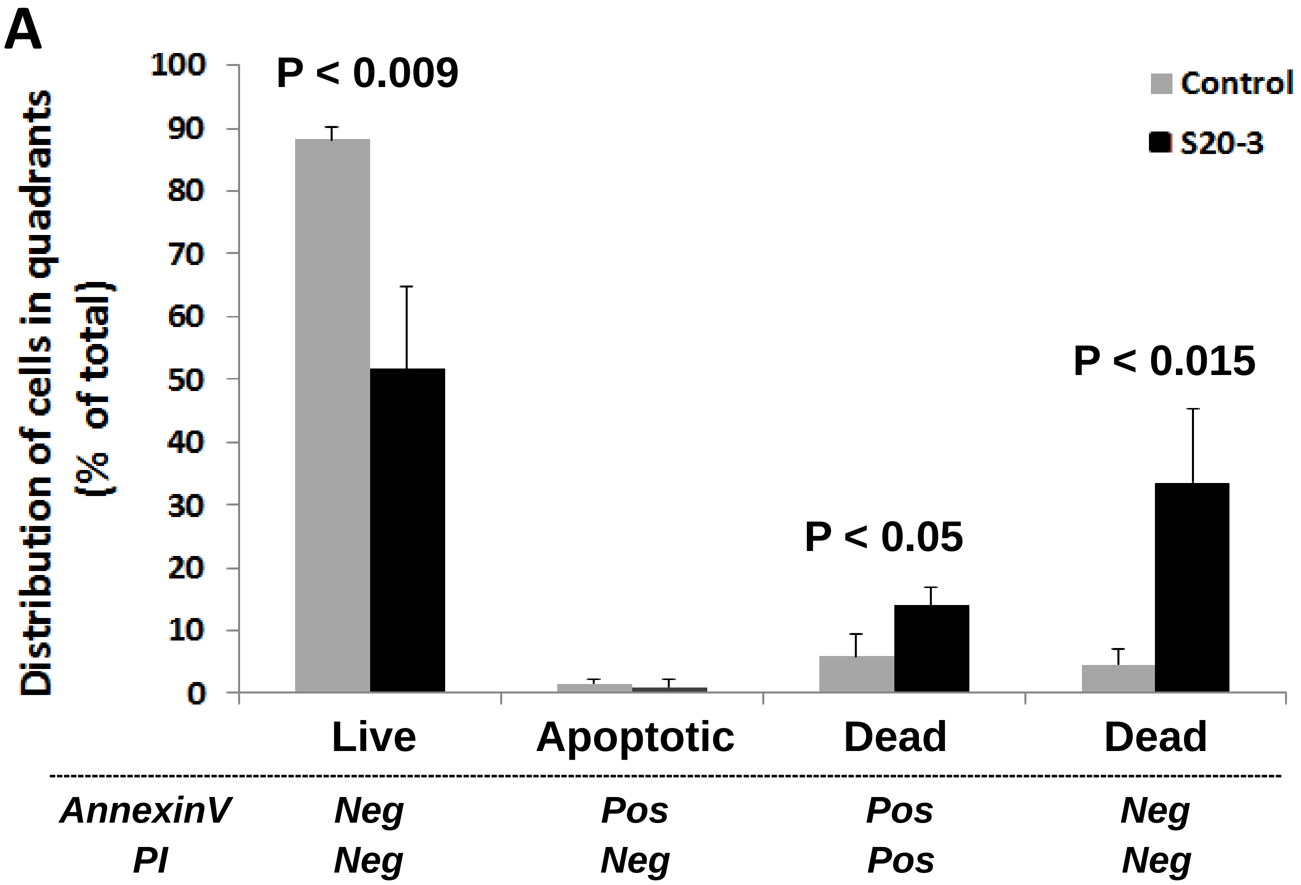
A
Distribution of cells in quadrants (% of total)
100
90
80
70
60
50
40
30
20
10
0
P < 0.009
P < 0.05
P < 0.015
Control
S20-3
Live
Apoptotic
Dead
Dead
AnnexinV
PI
Neg
Neg
Pos
Neg
Pos
Pos
Neg
Neg

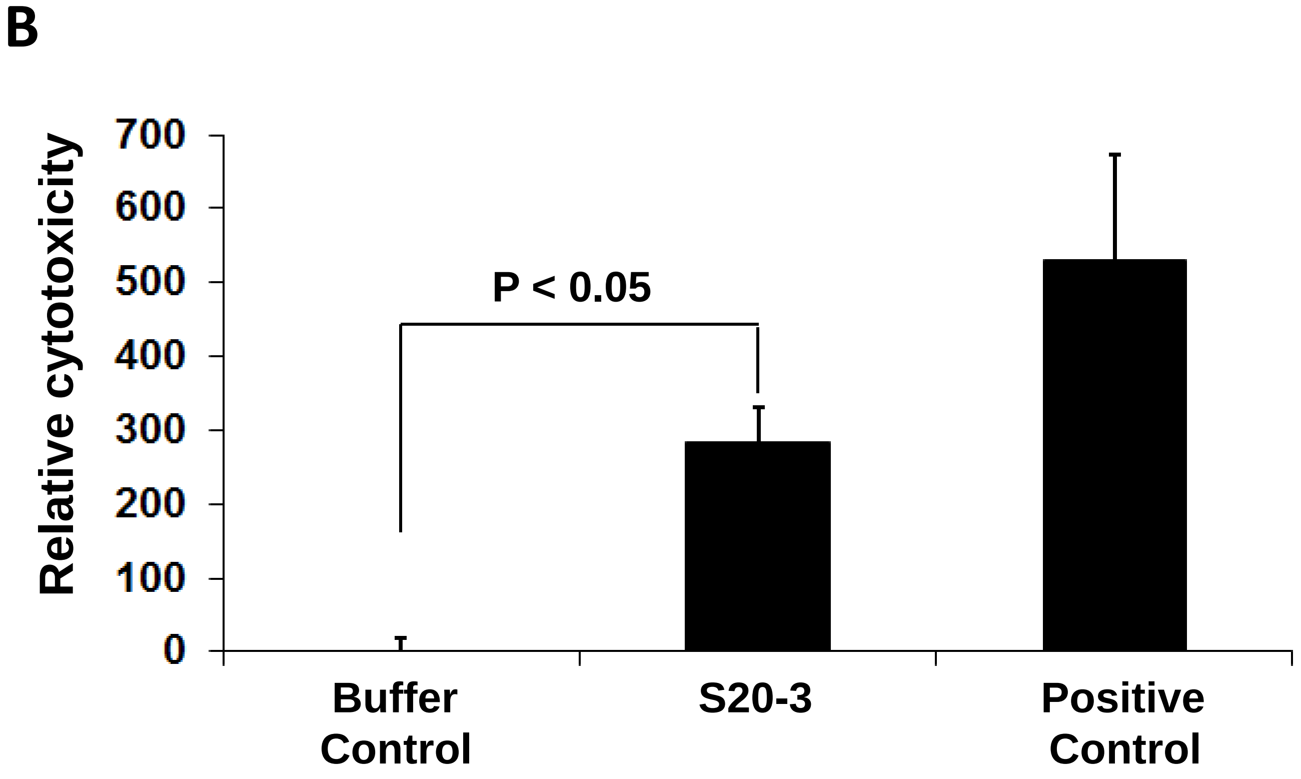
B
Relative cytotoxicity
700
600
500
400
300
200
100
0
P < 0.05
Buffer Control
S20-3
Positive Control
